# Genetic Evolution and Molecular Characterization of PRRSV GP5 in Germany

**DOI:** 10.3390/vetsci13070682

**Published:** 2026-07-13

**Authors:** Jiankun Pang, Qipeng Zhang, Chen Lv, Huawei Li, Xuyong Zhao, Ruining Wang, Keshan Zhang, Yaqiong Ye, Mengmeng Zhao

**Affiliations:** 1 Guangdong Provincial Key Laboratory of Animal Molecular Design and Precise Breeding, School of Animal Science and Technology, Foshan University, Foshan 528225, China; 20220420221@stu.fosu.edu.cn (J.P.); zhangqp1236@163.com (Q.Z.); lc0214fosu@163.com (C.L.); zks009@126.com (K.Z.); 2 Institute of Animal Product Quality and Safety Technology, Henan University of Animal Husbandry and Economy, Zhengzhou 450046, China; centrosome@126.com (H.L.); zhaoxy6868@163.com (X.Z.); 80882@hnuahe.edu.cn (R.W.)

**Keywords:** porcine reproductive and respiratory syndrome virus, Germany, GP5 gene, genetic variation, phylogeny, recombination

## Abstract

Porcine reproductive and respiratory syndrome (PRRS) has been prevalent in Germany for over 30 years, posing a significant threat to the local swine industry. There are limited analyses to offer German PRRSV ORF5 genetic and evolutionary characteristics and GP5 structural and functional features. In this study, a total of 518 sequences of the PRRSV GP5 gene were obtained from the GenBank database, encompassing 102 sequences from Germany to investigate the genetic relationships and GP5 structural and functional features. Similarly, phylogenetic and recombination analyses were used to identify genetic relationships. Two German PRRSV-1 lineage 1 strains exhibited more than 98.0% nucleotide similarity with vaccine strains. Eighteen German PRRSV-2 lineage 5 strains exhibited more than 98.0% nucleotide similarity with a prototype vaccine strain. German PRRSV-1 lineage 1 was predominant. GP5 structural and functional features were determined by N-glycosylation sites, B-cell epitopes, and transmembrane domain predictions. The main N-glycosylation patterns and transmembrane domains of German PRRSV-1 lineage 1 were time-dependent. Mutations of German PRRSV-1 lineage 3 in the primary neutralizing epitope were detected. In conclusion, these findings reveal lineage-specific molecular variation and improve the understanding of the molecular evolution of German PRRSV.

## Simple Summary

Porcine reproductive and respiratory virus (PRRSV) causes significant economic losses in the German swine industry. Yet data on its main structural protein, GP5, have not been systematically analyzed in Germany. GP5 is a glycosylated envelope protein, which plays an important role in immune evasion. In the present study, the evolutionary and structural characteristics of the GP5 gene were analyzed to predict potential evolutionary patterns of PRRSV. The analysis provides molecular insights into prevention strategies and vaccine development. Additional studies on whole genome analysis and spatial structure analysis are needed to deepen the understanding of German PRRSV evolution and the structure of GP5, which can facilitate vaccine development and lay the foundation for prevention strategies.

## 1. Introduction

Porcine reproductive and respiratory syndrome (PRRS) is caused by the porcine reproductive and respiratory syndrome virus (PRRSV). It is characterized by reproductive dysfunction in sows and respiratory symptoms in pigs of all ages. PRRSV is characterized as a small enveloped virus (50–65 nm) with a single-stranded positive-sense RNA genome [1]. Due to substantial genetic (50–60% similarity) and antigenic distinctions between European and North American isolates, PRRSV isolates have been categorized as the North American type (PRRSV-2) and the European type (PRRSV-1) [2]. Although PRRSV is endemic worldwide [3], it remains difficult to control. Since its emergence, the disease has caused significant economic losses to the global pig industry [4,5], as PRRSV undergoes evolution, immune evasion, and virulence changes through glycosylation shielding, antibody-dependent enhancement (ADE), recombination, immune decoys, and other strategies [6,7,8]. Although the German PRRSV genetic relationships have already been revealed by Greiser-Wilke et al. [9], the lineage classification and structural and functional features of German PRRSV still remain unknown, which handicaps the research on German PRRSV.

The GP5 protein is a glycosylated envelope protein encoded by the PRRSV ORF5 gene, and a major structural protein of the viral envelope. GP5 interacts with the M envelope protein to form heterodimers on the surface of viral particles [1]. Given its surface exposure, role in host cell attachment, and presence of the neutralizing epitope (NE), GP5 is subject to strong immune selection pressure and is therefore a prime target for novel vaccine development [10]. While NSP2 and GP3 exhibit considerable variability, GP5 demonstrates the greatest degree of variation in PRRSV and is therefore frequently employed in analyses of genetic diversity [10]. Although whole-genome analysis is widely used in studies of viral evolution and recombination [11,12], it generally requires more extensive sequence datasets. Therefore, GP5-based analysis was used to characterize the evolutionary relationships and genetic variation of the analyzed strains.

Germany, one of the core hubs of the European swine industry, plays an important role in cross-border transmission of PRRSV in Europe. Since the identification of PRRSV, PRRS has been prevalent in Germany for over 30 years, causing a significant economic impact locally [13]. Currently, the control of PRRSV in Germany primarily targets individual farms, whereas other countries have adopted national-level control strategies [14,15]. This study aimed to elucidate the genetic relationships and GP5 structural and functional features via genetic and protein analyses. These findings can provide a basis for the prevention and control of PRRS outbreaks and PRRSV vaccine development.

## 2. Materials and Methods

### 2.1. Dataset

To reveal the prevalence of PRRSV in Germany and assess the genetic recombination probability, a total of 518 sequences including 102 from Germany were collected from the GenBank database. Specifically, the remaining 416 sequences were sourced as follows: 64 from each of the Netherlands, Spain and Italy, 57 from Denmark, 44 from Poland, 21 from Hungary, 42 from the United States, 35 from China, and 25 reference sequences (Table S1).The inclusion of strains from other countries was warranted, as Germany is a major live pig exporter in Europe with significant PRRSV-2 presence [9,16,17,18].

A total of 102 German PRRSV strains were included in this study. Among them, 86 strains with available sampling locations were used for geographic distribution analysis, whereas the sampling locations of the remaining 16 strains were unavailable in the GenBank records.

For ascertaining the similarity and the features of German PRRSV GP5, 90 sequences were selected from the 518 sequences, comprising 50 German PRRSV-1 strains, 23 German PRRSV-2 strains, and 17 reference strains. The German strains were grouped based on their isolation years. This was done to ensure temporal diversity among German isolates and reduce dataset redundancy. When multiple GP5 sequences from the same year exhibited identity or high similarity, representative sequences were retained to avoid overrepresentation of closely related isolates. This procedure ensured that all sampling years remained represented in the structural and sequence analyses, resulting in a final dataset of 90 German GP5 sequences (Table S2).

### 2.2. German Geographic Distribution Analysis

The geographic information of 86 German PRRSV strains was retrieved from the corresponding GenBank records. The sampling locations were standardized according to the German federal states. Because Lower Saxony and North Rhine-Westphalia could not be consistently distinguished in several GenBank records, strains originating from these two federal states were grouped as northwest Germany for geographic analysis. Geographic distribution maps were generated using Python 3.6 software.

### 2.3. GP5 Sequence Similarity and Alignment Analyses

The 90 GP5 sequences were chosen for similarity and alignment analysis. Firstly, the Clustal W method in DNASTAR software (MegAlign Lasergene Version 7.0; DNASTAR, Inc., Madison, WI, USA) was used for alignment. Nucleotide similarity analysis was conducted based on the alignment results. Then the nucleotide sequences were converted into amino acid sequences using DNASTAR software. Subsequently, amino acid sequence similarity analysis was performed using the Clustal W method. Eventually, the alignment of the PRRSV-1 and PRRSV-2 GP5 amino acid sequences was analyzed.

### 2.4. Prediction of GP5 Structural and Functional Features

The translated amino acid sequences of the 90 GP5 sequences were used for GP5 structural and functional feature prediction. Initially, N-glycosylation site prediction was performed via the NetNGlyc server (version 1.0, http://www.cbs.dtu.dk/services/NetNGlyc/, accessed on 23 March 2026). Following the recommendations of the NetNGlyc server, a prediction score of 0.5 was used as the default cutoff to classify a site as glycosylated, while a more stringent threshold of 0.75 was applied to define high-confidence predictions [19]. Sites predicted as below the 0.5 threshold were classified as non-glycosylated according to the software output. Next, B-cell linear epitopes of the 90 GP5 sequences were predicted using BepiPred-3.0 software (version 3.0, Technical University of Denmark, Kongens Lyngby, Denmark). The default threshold of 0.1512, as recommended by the developers of BepiPred-3.0, was used for linear B-cell epitope prediction [20]. Finally, transmembrane domain prediction of the 90 GP5 sequences was performed using the DeepTMHMM 1.0 online server (https://services.healthtech.dtu.dk/services/DeepTMHMM-1.0/, accessed on 24 March 2026).

### 2.5. Phylogenetic and Recombination Analyses

A total of 518 GP5 sequences were used for phylogenetic tree reconstruction and genetic recombination analysis. Initially, the GP5 nucleotide sequences were aligned using the ClustalW algorithm implemented in MEGA software (version 7.0.26, Mega Limited, Auckland, New Zealand). To ensure comparable sequence coverage and preserve codon integrity across all isolates, ambiguously aligned positions, terminal gaps, and insertion/deletion regions were manually trimmed. The resulting codon-consistent alignment was subsequently used for phylogenetic and recombination analyses. Phylogenetic trees were inferred using both neighbor-joining (NJ) and maximum likelihood (ML) approaches, each with 1000 bootstrap replicates. The ML tree was constructed under the Kimura 2-parameter model with gamma-distributed rate variation and a proportion of invariant sites. All other settings followed default parameters. After the trees were generated, they were exported to the Interactive Tree of Life (iTOL) online platform for annotation. Potential recombination events in the GP5 gene among the 518 sequences were identified using seven algorithms integrated into the Recombination Detection Program (RDP) software (version 4.0): RDP, MaxChi, GeneConv, BootScan, SiScan, 3Seq, and Chimaera. Potential recombination events were confirmed only when supported by three or more of these algorithms, with a significance threshold set at *p* < 0.05. Furthermore, the identified recombination events were further validated using SimPlot software (version 3.5.1).

## 3. Results

### 3.1. Geographic Distribution Analysis of German PRRSV Strains

**Figure 1:**
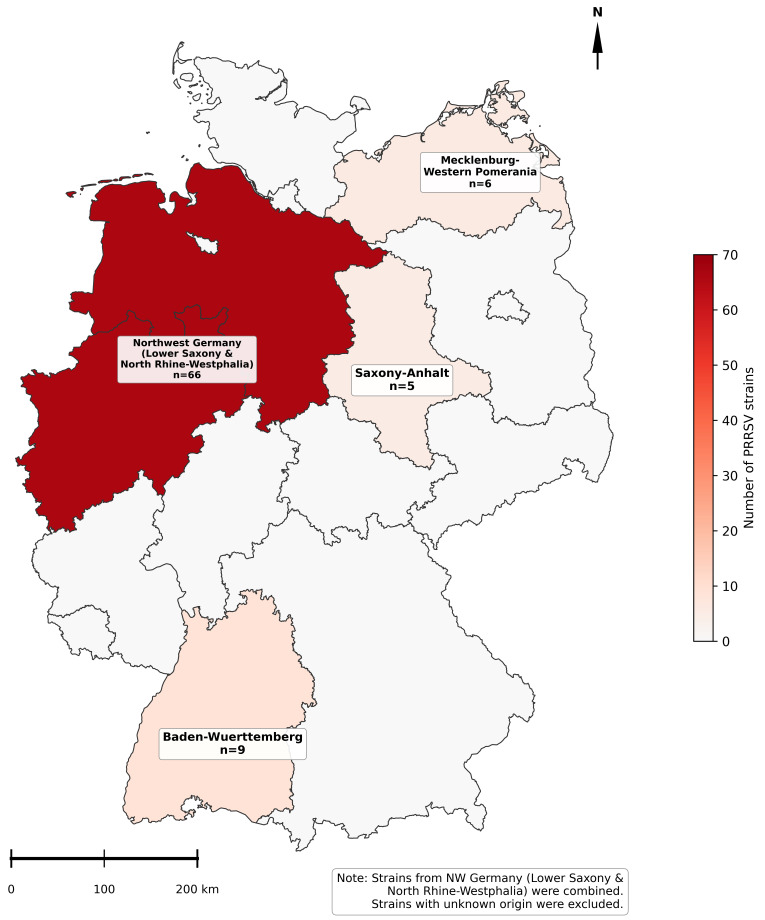
Geographic distribution analysis of the GP5 of German PRRSV 86 strains.

Among the 86 strains with available geographic information, most originated from northwest Germany (Lower Saxony and North Rhine-Westphalia), whereas comparatively few were collected from Baden-Württemberg, Mecklenburg-Vorpommern, and Saxony-Anhalt (Figure 1).

### 3.2. Similarity Analysis of German GP5 Sequences

Overall, nucleotide similarity among the 90 strains ranged from 60.6% to 100.0%, while amino acid similarity ranged from 51.3% to 100.0% (Table 1). PRRSV-2 lineage 5 had the highest within-lineage similarities, ranging from 90.0% to 100.0% (nucleotide) and 88.0% to 100.0% (amino acid), respectively. By contrast, PRRSV-1 lineage 3 showed the lowest within-lineage similarities, with nucleotide and amino acid similarity ranging from 85.3% to 100.0% and 86.1% to 100.0%, respectively. For PRRSV-2 lineage 5, except for the highest within-lineage similarities and the similarities between lineage 5 and reference strains, all maximum and minimum nucleotide similarities were at least 2% higher than the corresponding amino acid similarity.

For PRRSV-1 lineage 1, nucleotide and amino acid similarities with vaccine strains were 85.1–98.0% and 81.1–97.5%, respectively; two lineage 1 strains shared >98% nucleotide similarity with Amervac_PRRS-Spain-2009 and Porcilis_DV-MLV-Denmark-2014, respectively. The highest similarity between lineage 1 strains and the 94881-USA-2006 strain was 97.4% (nucleotide) and 94.6% (amino acid). The strains showing the lowest nucleotide similarity to each of the three vaccine strains were mostly different. For the classical Lelystad-Netherlands-1991 strain, nucleotide similarity with lineage 1 strains was 87.3–98.7%, with the highest in 2.72-Germany-1993 and Cresa3266-Germany-1996, and the lowest in V1662_22-Germany-2022. All nucleotide similarities between PRRSV-1 lineage 3 and reference strains were below 98.0%. Notably, 18 PRRSV-2 lineage 5 strains shared >98.0% nucleotide similarity with the prototype vaccine strain ATCC_VR-2332-USA-1995.

### 3.3. Amino Acid Sequence Alignment of German GP5 Sequences

**Figure 2:**
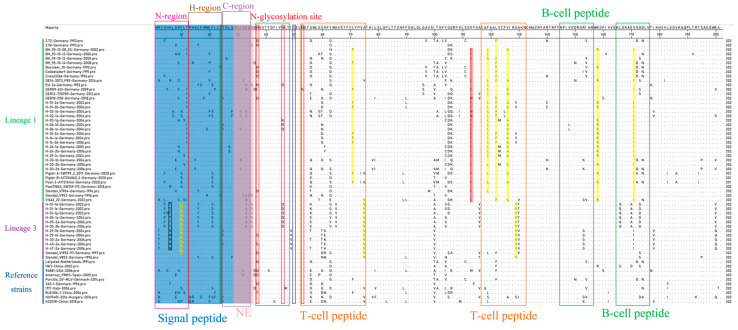
Amino acid sequence alignment of the GP5 of German PRRSV-1 60 strains. Annotations in the alignment are defined as follows: the signal peptide is labeled in blue, lineage-specific mutation sites are highlighted in yellow, amino acid position 24 is marked in green, the NE motif is indicated in pink, and amino acid position 113 is displayed in red. For boxed annotations, the N-region, H-region and C-region are enclosed in pink, brown and purple boxes, respectively; N-glycosylation sites, B-cell epitopes and T-cell epitopes are framed in red, green and orange boxes, respectively; amino acid position 6 is marked with a black box, and amino acid position 50 is marked with a blue box.

Sequence alignment was performed using the MegAlign module of DNASTAR software. This alignment detected amino acid mutations in PRRSV-1 GP5 sequences (Figure 2/Figure S1), with many located in the signal peptide. Abundant lineage-specific mutations in lineage 1 and lineage 3 were observed. However, some mutations in German PRRSV-1 were consistent with those in reference strains. Specifically, the lineage 1-specific V171→I171 mutation was in the B-cell epitope, which was absent in all reference strains.

In the N-region of the signal peptide, most lineage 3 strains harbored two mutations at amino acid 6, along with an additional lineage 3-specific mutation. Amino acid 24 was highly conserved, with one mutation detected across 60 PRRSV-1 sequences: C24→P24 in Boxmeer_10-Germany-1992. The neutralizing epitope (NE) region was relatively conserved compared with other regions, but some strains (especially lineage 3 strains) carried mutations distinct from those in reference strains. Notably, high-frequency mutations occurred at the 37th N-glycosylation site. Mutations at amino acid 113 were located in the transmembrane domain (TMD).

Turning to sequence differences between modified live vaccine (MLV) strains and strains with >98.0% nucleotide similarity to MLV strains, characteristic mutations were located in the N-region and H-region of the signal peptide in these high-similarity strains. For the classical strain Lelystad-Netherlands-1991 and its paired high-similarity strains 2.72-Germany-1993 and Cresa3266-Germany-1996, the characteristic mutation was also located in the N-region of the signal peptide.

**Figure 3:**
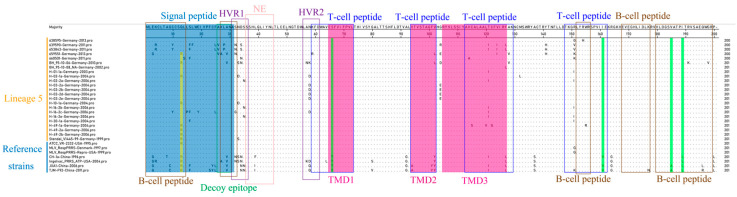
Amino acid sequence alignment of the GP5 of German PRRSV-2 30 strains. Color-coded annotations in the alignment are specified as follows: the signal peptide is indicated in blue, amino acid residue at position 13 is highlighted in yellow, geographically specific conserved regions of the strains are marked in green, and the TMDs are displayed in red. For boxed annotations: B-cell epitopes are enclosed in brown boxes, decoy epitopes in green boxes, hypervariable regions in purple boxes, the NE motif in pink boxes, and T-cell epitopes in blue boxes.

Sequence alignment of PRRSV-2 GP5 strains revealed amino acid mutations, primarily concentrated in the signal peptide, hypervariable region 1 (HVR1) and hypervariable region 2 (HVR2) (Figure 3/Figure S2). Mutations at amino acid 13 were detected in most strains. Lineage 5 strains exhibited no mutations in the NE, a pattern distinct from that observed in reference strains.

### 3.4. N-Glycosylation Site Prediction of German GP5 Sequences

Five potential N-glycosylation sites (N33, N35, N37, N46, and N53) were identified in PRRSV-1 via the NetNGlyc server (Table 2). A total of seven distinct N-glycosylation patterns were summarized across all time periods. The dominant N-glycosylation pattern of lineage 1 before 2001 was distinct from that after 2001. The dominant pattern before 2001 was N46 and N53, which aligned with that of Lelystad-Netherlands-1991. However, the dominant pattern after 2001 was N37, N46, and N53, which was consistent with that of Porcilis_DV-MLV-Denmark-2014. Overall, the most prevalent N-glycosylation pattern of lineage 1 across all time periods was still N37, N46, and N53. Moreover, none of the lineage 1 strains exhibited the N35, N46, and N53 glycosylation pattern seen in Amervac_PRRS-Spain-2009. As for lineage 3, the dominant N-glycosylation pattern was N37 and N53, which differed from that of lineage 1. Lineage 3 showed diverse N-glycosylation patterns; one pattern had a glycosylation confidence score below the default 0.5 threshold at the N53 site. Moreover, only three lineage 5 strains in 2006 exhibited the glycosylation pattern matching that of HP-PRRSV strains. The numbers of available sequences varied considerably among sampling years, with relatively more sequences available for 2004–2006 than for other years.

In comparison with PRRSV-1, PRRSV-2 exhibited more diverse N-glycosylation patterns and sites (N30, N32, N33, N34, N35, N44, and N51) (Table 3). The dominant N-glycosylation pattern of lineage 5 did not change over time. Regardless of the time period, most strains harbored the N30, N33, N44, and N51 glycosylation pattern, which was consistent with that of ATCC_VR-2332-USA-1995; however, fewer than 18 strains presented this pattern. Moreover, only three lineage 5 strains in 2006 exhibited the glycosylation pattern matching that of HP-PRRSV strains.

### 3.5. B-Cell Epitopes Prediction of German GP5 Sequences

**Figure 4:**
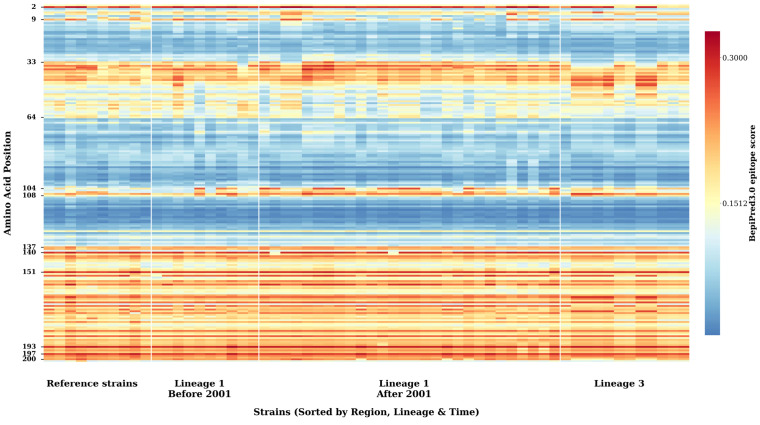
B-cell epitope prediction of German 60 PRRSV-1 GP5 using BepiPred-3.0 software.

The B-cell epitopes were predicted by the BepiPred-3.0 software (Figure 4). Generally, the B-cell epitopes predominantly resided in four specific regions: amino acids 1–9 33–64, 104–108, and 137–200. The top-ranking sites based on general scores were amino acids 2, 140, 151, 193, and 197. Unlike the N-glycosylation patterns and TMDs in lineage 1, the B-cell epitopes of lineage 1 did not change over time. Also, there were no differences among lineage 1 strains, lineage 3 strains and reference strains in B-cell epitopes.

**Figure 5:**
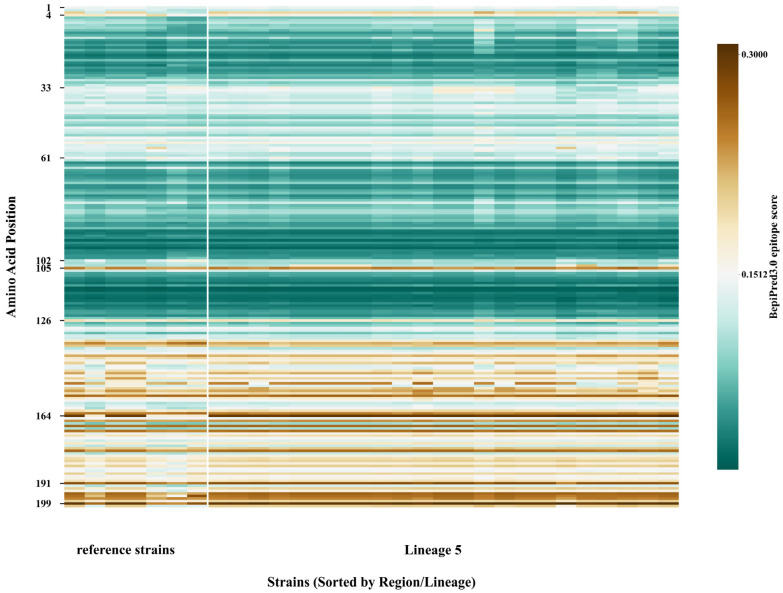
B-cell epitope prediction of German 30 PRRSV-2 GP5 using BepiPred-3.0 software.

Similar to PRRSV-1 B-cell epitopes, the PRRSV-2 strains showed that the B-cell epitopes were primarily concentrated in four major regions: amino acids at positions 1–4, 33–61, 102–105, and 126–200 (Figure 5). No changes were observed in the overall regions of B-cell epitopes between the reference strains and lineage 5 strains. Moreover, the sites with the highest overall scores were at amino acid positions 164, 191, and 199.

### 3.6. Transmembrane Domain Prediction of German GP5 Sequences

**Figure 6:**
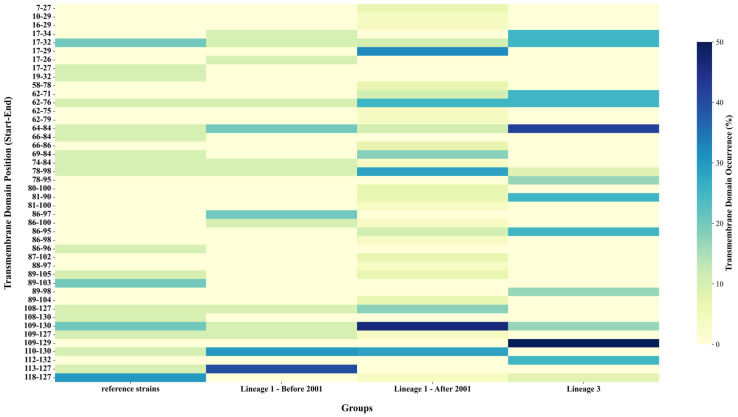
Transmembrane domain prediction of German 60 PRRSV-1 GP5 using the DeepTMHMM 1.0 online server.

A clustered distribution pattern of PRRSV-1 TMDs was identified via DeepTMHMM 1.0 (Figure 6). TMDs were primarily situated at amino acid positions 7–32, 58–105, and 108–132. The highest-occurrence TMDs of lineage 1 before 2001 were distinct from those after 2001. Moreover, the high-occurrence TMDs in lineage 1, including 109–130aa, 110–130aa, and 113–127aa, were longer than the highest-occurrence TMDs in reference strains (118–127aa). The high-occurrence lineage 1 TMDs (109–130aa, 110–130aa, and 113–127aa) differed from the high-occurrence lineage 3 TMDs (109–129aa and 112–132aa). This pattern was also observed in lineage 3: its TMDs (109–129aa and 112–132aa) were longer than the reference TMDs located at 118–127aa.

**Figure 7:**
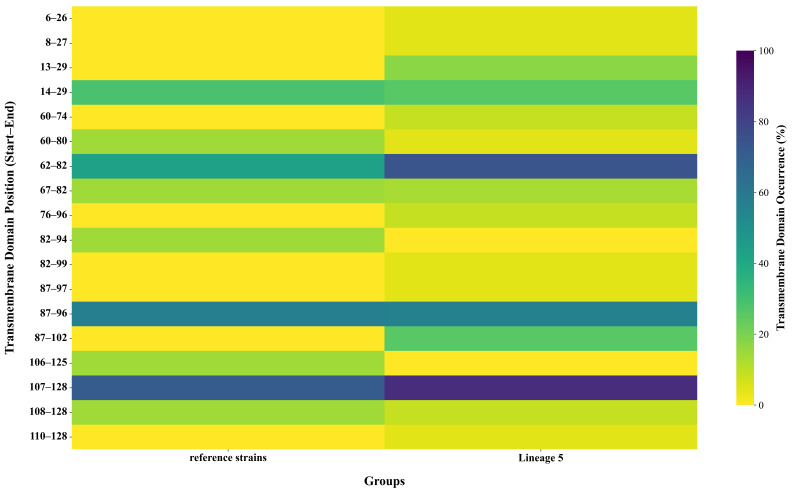
Transmembrane domain prediction of German 30 PRRSV-2 GP5 using the DeepTMHMM 1.0 online server.

In line with PRRSV-1 TMDs, PRRSV-2 TMDs also exhibited a clustered distribution pattern (Figure 7). TMDs were clustered around amino acid positions 6–29, 60–102, and 106–128. The high-occurrence TMDs of lineage 5 (62–82aa, 87–96aa, and 107–128aa) aligned with the high-occurrence TMDs of reference strains. Moreover, several TMDs (6–26aa, 8–27aa, and 13–29aa) were detected, which were not present in the reference strains.

### 3.7. Phylogenetic Analysis of German GP5 Sequences

**Figure 8:**
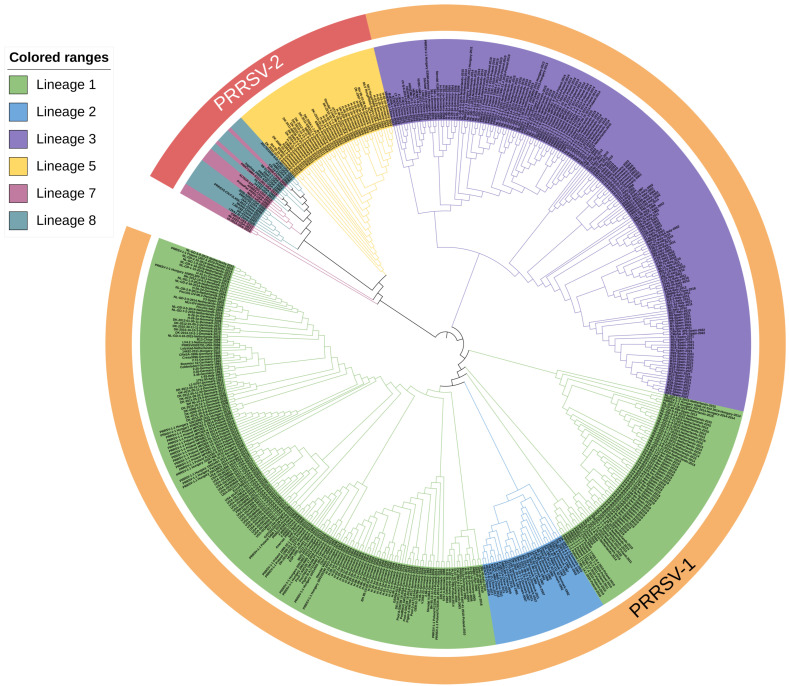
Phylogenetic analysis of the GP5 gene derived from 518 PRRSV strains. This phylogenetic tree was built using the NJ algorithm in MEGA software, and bootstrap analysis with 1000 replicates was performed to verify the topological stability of the resulting tree.

**Figure 9:**
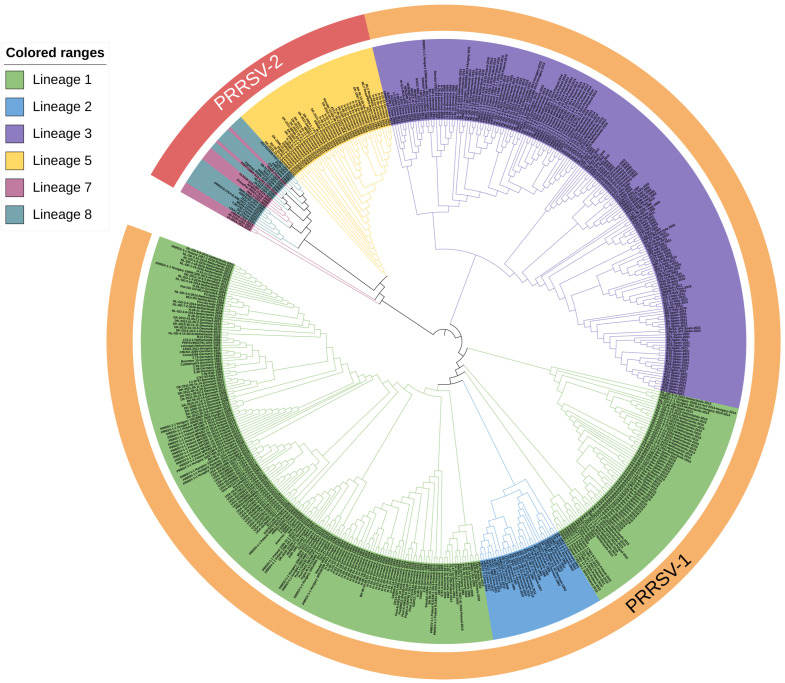
Phylogenetic analysis of the GP5 gene from 518 PRRSV strains. This phylogenetic tree was built in MEGA using the ML algorithm based on the K2 + G + I model, and bootstrap analysis with 1000 replicates was performed to validate the topological stability of the resulting tree.

Phylogenetic analysis of the 518 PRRSV GP5 gene sequences was performed using MEGA software (Figure 8 and Figure 9, Figures S3 and S4). The 518 PRRSV strains were classified into six lineages. All PRRSV-1 strains within the 518 sequences were categorized as subtype 1. All German PRRSV-1 strains collected in this study were located in lineage 1 and lineage 3. German PRRSV-1 lineage 1 was in a dominant position with a total of 63 strains. The German PRRSV-1 lineage 1 strains showed clustered distribution. All German PRRSV-2 strains collected in this study clustered within lineage 5.

### 3.8. Recombination Analysis of German GP5 Sequences

A total of 518 isolates were analyzed using RDP 4.0 (Table 4). However, no recombination event involving German strains was supported by more than three methods with *p*-values < 0.05. Recombination event 1 was detected by only two methods and therefore did not meet the predefined criteria for a reliable recombination event. In addition, no minor parental strain could be identified for this event.

## 4. Discussion

The results revealed the PRRSV prevalence in Germany based on the GP5 gene and its partial features, indicating mutations and genetic variation that pose potential risks to the swine industry. The predominance of strains from northwest Germany is consistent with the high density of pig farms in this region. Previous studies by David et al. and Nathues et al. reported that northwest Germany has one of the highest pig farm densities in the country, whereas pig farm density is comparatively lower in Saxony [21,22]. Nevertheless, because the available sequences were unevenly distributed across regions, the observed geographic pattern may have been influenced by sampling bias and therefore should be interpreted with caution.

For phylogenetic analysis, frequent live pig exchange within Europe may facilitate viral recombination, potentially generating intermediate sequences that could obscure phylogenetic boundaries. This process may partially explain why no further subdivision into clades was observed in the present analysis.

In PRRSV-1, the predominance of lineage 1 aligns with the model proposed by Balka et al. [23], in which the ancestors of PRRSV-1 subtype 1 originated in Eastern Europe, entered Germany via wild boar, and then spread and diversified with Germany as a hub. Germany may have contributed to the regional dissemination of lineage 1 because of its extensive live pig trade, which is one of the major exporters of live pigs in Europe. The high prevalence of lineage 1 in Germany may be associated with multiple factors, including its pig imports (predominantly from the Netherlands), as well as the application of lineage 1 vaccines.

However, recombination analysis based on the GP5 gene did not detect confident recombination events. Therefore, while recombination or other evolutionary processes may have contributed to genetic diversity in German PRRSV-1, such hypotheses cannot be confirmed using ORF5-based analyses alone and require whole-genome data for validation. Moreover, the unknown minor parent reported by RDP indicates that no suitable minor parental sequence was identified among the analyzed sequences, which may reflect incomplete sampling or the absence of the true parental sequence from the current dataset.

For PRRSV-1 lineage 1, several German strains showed high nucleotide similarity (>98%) to vaccine strains, suggesting a close genetic relationship at the GP5 gene level. According to Collins et al. [24], such similarity may indicate relatedness but it is not sufficient to infer evolutionary mechanisms or strain origins. A strain showing 97.4% similarity (94881-USA-2006) may also demonstrate a relatively close genetic relationship to German lineage 1 strains. In addition, differences in the degree of similarity to various vaccine strains suggest heterogeneous genetic distances among strains, reflecting complex evolutionary patterns. More recently isolated strains showed greater genetic distance from the Lelystad strain, which may reflect continuous viral evolution over time.

The available data suggest that lineage 3 may have been introduced into Germany through cross-border movement of pigs or related trade activities, although its introduction route cannot be inferred from GP5 sequence data alone. No lineage 3 strains were detected after 2006; however, this observation may have been influenced by limited sequence availability and uneven sampling across Europe. The absence of closely related reference strains suggests that the evolutionary origin of German lineage 3 cannot be readily inferred from the currently available reference sequences.

In addition to PRRSV-1, Germany primarily imports pigs from Denmark, where a PRRSV-2 epidemic linked to the use of a PRRSV-2 vaccine has been described by Madsen et al. [25]. Although epidemiological links exist between Denmark and Germany, the available GP5 sequence data do not allow the geographic origin of German PRRSV-2 strains to be inferred. Therefore, Denmark can only be considered as one of several potential sources rather than a confirmed origin. Eighteen strains shared >98.0% nucleotide similarity with ATCC_VR-2332-USA-1995, indicating a close genetic relationship to vaccine strains. Furthermore, the highest within-lineage similarity was observed in lineage 5, suggesting relatively limited genetic divergence within this lineage. However, nucleotide similarity alone does not provide evidence for biological characteristics such as pathogenicity or virulence. The phenomenon where the nucleotide similarity is higher than the amino acid similarity in lineage 5 may be attributed to nonsynonymous mutations. These mutations may reflect ongoing genetic diversification of lineage 5 strains.

The signal peptide mediates the initial targeting of GP5 to the rough endoplasmic reticulum (ER), with subsequent translocation of the protein into the ER lumen via a hetero-oligomeric membrane channel complex [26]. Given the established role of the signal peptide in GP5 maturation, the substitutions of PRRSV-1 in signal peptide may have functional implications, although their biological significance remains to be experimentally determined. Similar amino acid substitutions observed in both German and reference strains may reflect similar selective pressures, although alternative evolutionary scenarios cannot be excluded. Numerous lineage-specific mutations may have potential relevance to lineage classification. These lineage-specific mutations may provide useful molecular markers for lineage discrimination and may reflect genetic divergence among lineages.

The 33–64 aa region of German PRRSV-1 GP5 and the 33–61aa region of German PRRSV-2 GP5 appear to be immunologically important, as predicted N-glycosylation sites were consistently located within these regions. This colocalization suggests that glycan shielding may play a role in modulating antibody accessibility. Consistently, Vu et al. and Thaa et al. have reported that, with the decoy epitope sequence eliminated by most wild strains during signal peptide cleavage, glycosylation shielding may contribute to delayed neutralizing antibody responses [27,28]. However, high-scoring predicted B-cell epitope sites such as amino acid positions 151 in PRRSV-1 and 164 in PRRSV-2 may not be located within the major extracellular regions identified in this study but may instead be located within the cytoplasmic domain. Therefore, their potential immunological relevance remains uncertain and warrants further experimental investigation.

For German lineage 1 strains, the C24→P24 substitution plays an important role. Wissink et al. have reported that this substitution is associated with enhanced signal peptide cleavage efficiency, altered N-glycosylation, and increased exposure of the NE [29]. In addition, substitutions at amino acid 24 may influence neutralizing antibody recognition and signal peptide processing, thereby affecting GP5 maturation and N-glycosylation [26,29].

The key mutations in the N-region and H-region of the signal peptide, identified in strains with >98.0% nucleotide similarity to MLV strains, may affect signal peptide function. In particular, reduced hydrophobicity of the H-region could potentially impair ER membrane translocation efficiency and influence GP5 processing [27]. Although the functional consequences of these substitutions remain unclear, Wei et al. reported associations between N-region mutations and virulence-related phenotypes, suggesting that these mutations warrant further investigation [30]. Overall, these signal peptide substitutions may contribute to phenotypic variation related to GP5 processing; however, their potential role in immune evasion remains to be experimentally validated. For TMD mutations at amino acid 113, these substitutions may influence the structural properties of the transmembrane domain, potentially affecting GP5-M interactions.

The first PRRSV-1 vaccine was introduced in Germany in 2001 [31], and shifts in the dominant glycosylation pattern may be associated with temporal changes following vaccine introduction, although other evolutionary forces cannot be excluded. Because sequence availability differed substantially among sampling years, the observed temporal shift should also be interpreted with caution, as uneven temporal sampling may have influenced the apparent distribution of glycosylation patterns. The emergence of N37 after 2001 may have biological relevance, as glycosylation at this position has been reported to mask critical B-cell epitopes [32]. Badaoui et al. reported that N37 may mask the NE and thereby reduce antibody accessibility [33]. This shift may contribute to altered antigenic properties and may be associated with the predominance of the N37-containing glycosylation pattern. The absence of strains with the N35, N46, and N53 pattern may be related to the possibility that N37 provides greater glycan shielding than N35 because of its closer proximity to the NE. However, this hypothesis requires experimental validation.

For B-cell epitope prediction results, the stability of the 33–64aa B-cell epitope in lineage 1 over time may be related to the limited variation of N-glycosylation sites. The stability of other predicted B-cell epitope regions may be explained by their structural locations: the 1–9aa region lies within the signal peptide, which is cleaved during GP5 maturation, while 104–108aa and 137–200aa were located in the cytoplasmic domain.

For PRRSV-1 lineage 3, amino acid 6 and lineage 3-specific mutations in the N-region may show similar patterns to N-region mutations observed in lineage 1. Notably, mutations within the NE region may alter its structural conformation, potentially affecting antigenic properties. The glycosylation pattern in the absence of the N46 site may also influence antigenic characteristics, although its biological significance remains unclear [34]. Low confidence scores (<0.5) for the N53 site across three strains, together with the diversity of glycosylation patterns in lineage 3, may reflect ongoing diversification of glycosylation. Although B-cell epitope regions appear conserved, this does not exclude the possibility of antigenic modulation through NE conformational changes, as suggested by alignment results.

With regard to German PRRSV-2, hypervariable regions (HVRs) have been reported to contribute to antigenic variation [35], suggesting that accumulated mutations may alter antigenic characteristics. Yu et al. have reported that mutations at amino acid 13 are associated with altered virulence [36]. Notably, no mutations were detected in the NE, which differs from patterns observed in reference strains. This observation may reflect differences in evolutionary pressures between Germany and regions where stronger vaccine-driven selection has been reported.

Furthermore, the dominant N-glycosylation pattern was similar to that of ATCC_VR-2332-USA-1995. This pattern may indicate potential genetic relatedness to vaccine strains. Although several lineage 5 strains show high sequence similarity to ATCC_VR-2332-USA-1995, variation in glycosylation patterns indicates continued evolution following divergence from a common ancestor. The glycosylation pattern observed in three lineage 5 strains was also consistent with that of HP-PRRSV strains, which may reflect either similar selective pressures or independent evolutionary events. In contrast, despite differences in sequence similarity between PRRSV-1 and PRRSV-2, B-cell epitope analysis indicated conserved overall structural features and core antigenic regions. No differences in predicted B-cell epitopes were observed between lineage 5 and reference strains, suggesting that key epitope features may be relatively conserved. As the present findings were based on computational predictions, their proposed functional implications remain hypothetical until confirmed by experimental studies.

For German lineage 1 strains, the temporal shift in the predominant TMD pattern observed before and after 2001 coincided with the introduction of PRRSV-1 vaccines in Germany. However, whether this temporal association reflects vaccine-related selective pressures or other evolutionary processes remains unclear. GP5 interacts with the M envelope protein through their TMDs to form heterodimers on the virion surface, facilitating efficient host cell entry [37]. The longer predicted TMDs of lineage 1 and lineage 3 may alter the structural organization of the GP5 transmembrane region, potentially affecting GP5-M interactions. The distinct predominant TMD patterns observed between lineage 1 and lineage 3 may reflect lineage-specific mutations.

With regard to PRRSV-2, the three clustered TMDs were consistent with the overall TMD organization observed in PRRSV-1, suggesting that TMD architecture is largely conserved across German PRRSV strains. Notably, the conservation of the predominant TMDs may reflect the limited genetic divergence between German lineage 5 strains and the reference strains. However, the emergence of additional TMDs among German strains may indicate ongoing genetic diversification within lineage 5.

Some questions remain unresolved. For example, the functional consequences of mutations within predicted T-cell epitopes remain unclear, as sequence-based analyses alone cannot determine their effects on antigen processing, MHC presentation, or T-cell recognition. In addition, whole-genome sequences were not included in this study, limiting a comprehensive assessment of the phylogenetic relationships and evolutionary history of German PRRSV strains. Furthermore, this study focused exclusively on the GP5 gene. Although GP5 is a major structural protein involved in viral evolution, antigenicity, and vaccine design, analyses based on a single gene cannot fully capture genome-wide evolutionary dynamics or the contributions of other viral proteins to virulence and immune evasion.

Nevertheless, by characterizing GP5 variation across German PRRSV strains, this study provides insights into molecular features associated with genetic evolution, antigenic variation, and virulence-related characteristics. These findings provide a foundation for future experimental validation and whole-genome investigations, and may contribute to PRRS surveillance, prevention, and vaccine development.

## 5. Conclusions

This study provides a comprehensive molecular characterization of the GP5 gene in German PRRSV-1 and PRRSV-2 strains. Geographically, most PRRSV strains were concentrated in northwest Germany. No recombination events were detected within the GP5 gene. German PRRSV-1 lineage 1 was identified as the predominant lineage. Predicted B-cell epitopes were highly conserved across different lineages, whereas lineage-specific mutations and temporal variation in the predominant N-glycosylation patterns and transmembrane domains were identified. In addition, several German PRRSV-1 and PRRSV-2 strains showed high sequence similarity to modified live vaccine strains. Most PRRSV strains were geographically concentrated in northwest Germany rather than evenly distributed across the country, suggesting a potential regional sampling bias or reflecting differences in pig population density.

Collectively, these findings demonstrate continued molecular diversification of German PRRSV while highlighting the conservation of key antigenic regions. The identified molecular characteristics provide insights into viral evolution and may improve the understanding of antigenic variation and the genetic diversity of German PRRSV strains. These findings support continued molecular surveillance of PRRSV and emphasize the value of integrating GP5 characterization with whole-genome sequencing and experimental validation to improve epidemiological monitoring and future vaccine development.

## Figures and Tables

**Table 1:** Similarity analysis of German 90 PRRSV GP5 strains.

			PRRSV-1		PRRSV-2	Reference Strains
			Lineage 1	Lineage 3	Lineage 5	
PRRSV-1	lineage 1	nt	82.7–**100.0**	81.5–90.9	61.1–66.2	60.9–98.7
		aa	82.2–**100.0**	82.2–92.6	**51.3**–58.9	52.8–98.0
	lineage 3	nt		85.3–**100.0**	61.0–63.6	61.6–91.3
		aa		86.1–**100.0**	51.8–56.3	53.3–92.6
PRRSV-2	lineage 5	nt			90.0–**100.0**	**60.6**–**100.0**
		aa			88.0–**100.0**	52.8–**100.0**
Reference strains		nt				**60.6**–**100.0**
		aa				54.3–**100.0**

Sequence similarity values are presented as ranges (minimum–maximum), with the minimum and maximum nucleotide and amino acid sequence similarities highlighted in bold.

**Table 2:** N-glycosylation site prediction of German 60 PRRSV-1 GP5.

		N-Glycosylation Site					Number of Sequence	% of Each Lineage
		33	35	37	46	53		
Reference strain	Lelystad				x	x		
	340-1				x	x		
	HK3			x	x	x		
	IT17			x	x	x		
	BJEU06-1		x		x	x		
	94881				x	x		
	Amervac_PRRS		x		x	x		
	Porcilis_DV-MLV			x	x	x		
	HU19401-2016			x	x	x		
	KZ2018			x	x	x		
lineage 1	1991				x	x	1	2.94%
	1992				x	x	1	2.94%
		x				x	1	2.94%
	1993				x	x	2	5.88%
	1996				x	x	2	5.88%
				x	x	x	2	5.88%
	2000			x	x	x	1	2.94%
	2002			x	x	x	1	2.94%
	2003			x	x	x	1	2.94%
	2004			x	x	x	5	14.71%
	2005			x	x	x	1	2.94%
	2006			x	x	x	7	20.59%
	2008			x	x	x	1	2.94%
	2009				x		1	2.94%
	2012			x	x	x	1	2.94%
	2014			x	x	x	1	2.94%
	2018				x	x	1	2.94%
	2020			x	x	x	3	8.82%
	2022			x	x	x	1	2.94%
lineage 3	1997				x	x	1	8.33%
	2003			x		x	3	25.00%
	2004			x		x	1	8.33%
				x	x	-	1	8.33%
			x		x	x	1	8.33%
	2006			x		x	2	16.67%
				x	x	-	2	16.67%
			x		x	x	1	8.33%

-: representing the potential N-glycosylation sites below the default threshold of 0.5; x: representing the potential N-glycosylation sites above the default threshold of 0.5.

**Table 3:** N-glycosylation site prediction of German 30 PRRSV-2 GP5.

		N-Glycosylation Site							Number of Sequence	% of Each Lineage
		30	32	33	34	35	44	51		
Reference strains	ATCC_VR-2332	x		x			xx	x		
	CH-1a				x		xx	x		
	Ingelvac_PRRS_ATP				x		xx	x		
	JXA1	x			-	x	xx	x		
	MLV_RespPRRS	x		x			xx	x		
	MLV_RespPRRS-Repro	x		x			xx	x		
	TJM-F92	x			-	x	xx	x		
lineage 5	1999	x					xx	x	1	4.55%
	2002	x		x			xx	x	1	4.55%
	2003	x		x			xx	x	1	4.55%
	2004	x					xx	x	2	9.09%
		x		x			xx	x	6	27.27%
	2006	x				x	xx	x	3	13.64%
		x		x			xx	x	2	9.09%
	2010	x			x		xx	x	1	4.55%
	2011		x	x			xx	x	2	9.09%
		x		x	x		xx	x	1	4.55%
	2013			x			xx	x	1	4.55%
		x		x			xx	x	1	4.55%

-: representing the potential N-glycosylation sites below the default threshold of 0.5; x: representing the potential N-glycosylation sites above the default threshold of 0.5; xx: representing the potential N-glycosylation sites above the threshold of 0.75.

**Table 4:** Recombination analysis of 518 PRRSV GP5 strains.

Recombination Event	Recombinant Strain (Lineage)	Main Parental Strain (Lineage)	Minor Parental Strain (Lineage)	Recombinant Breakpoint	Recombination Analysis Method
1	BH_95-10-08_EU-Germany-2002(1)	DK-2010-10-10-3-Denmark-2010(1)	unknown	196–294 (446-13)	RDP (*p* = NS) GENECONV (*p* = NS) BootScan (*p* = NS) MaxChi (*p* = 1.335 × 10^−2^) Chimaera (*p* = 1.570 × 10^−3^) SiScan (*p* = NS) 3Seq (*p* = NS)

NS: no significant *p*-value.

## Data Availability

The original contributions presented in this study are included in the article/Supplementary Material. Further inquiries can be directed to the corresponding authors.

## Supplementary Materials

The following supporting information can be downloaded at: https://www.mdpi.com/article/10.3390/vetsci13070682/s1, Figure S1: Amino acid sequence alignment of the GP5 of German PRRSV-1 60 strains; Figure S2: Amino acid sequence alignment of the GP5 of German PRRSV-2 30 strains; Figure S3: Phylogenetic tree of the GP5 gene from 518 PRRSV strains constructed using the NJ method in MEGA, with 1000 bootstrap replicates; Figure S4: Phylogenetic tree of the GP5 gene from 518 PRRSV strains inferred using the ML method in MEGA under the K2+G+I model, with 1000 bootstrap replicates; Table S1: Reference information for the GP5 sequence of 69 PRRSV-2 strains; Table S2: Reference information for the GP5 sequence of 35 PRRSV-2 strains.
