# Supplementary figures and images for: Genetic Evolution and Molecular Characterization of PRRSV GP5 in Germany
Source: Vet Sci. 2026 Jul 13;13(7):682. doi: 10.3390/vetsci13070682 (PMC13431494; doi:10.3390/vetsci13070682)

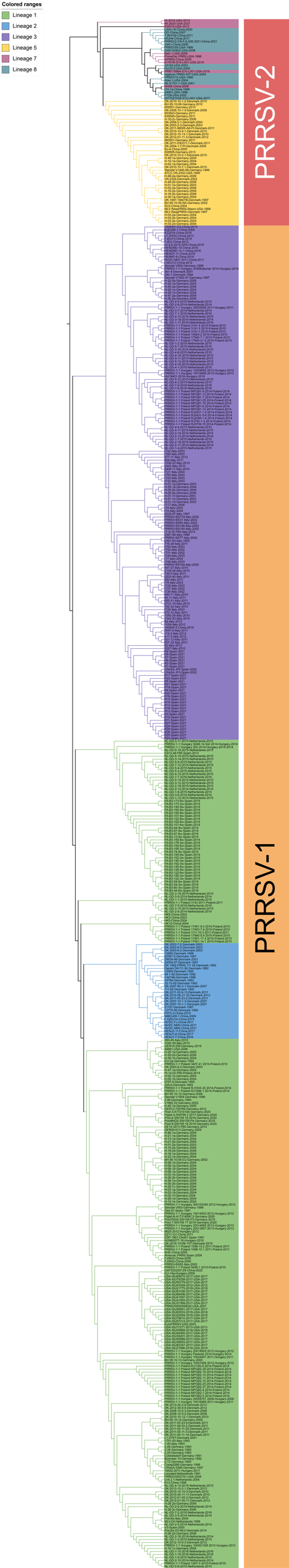

Supplement: Supplementary file 1 [file vetsci-13-00682-s001.zip › Fig. S3 Phylogenetic tree of the GP5 gene from 518 PRRSV strains constructed using the NJ method in MEGA, with 1000 bootstrap replicates.png]

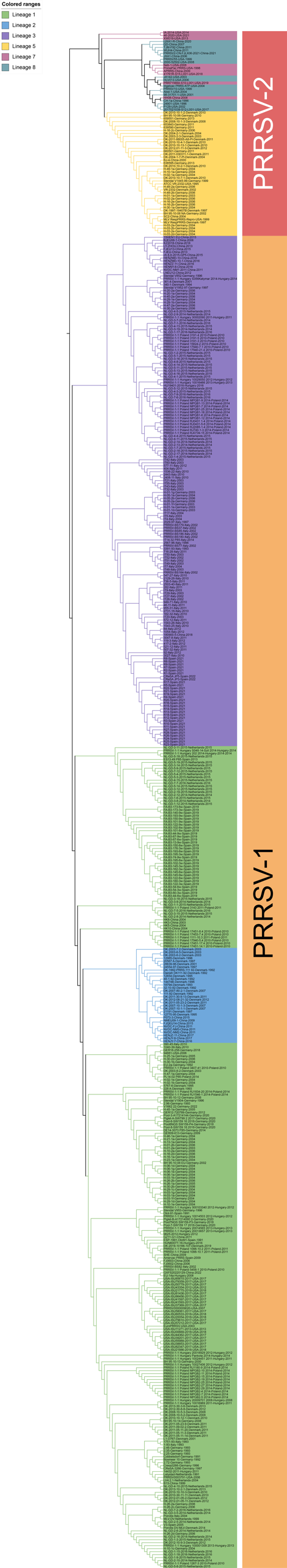

Supplement: Supplementary file 1 [file vetsci-13-00682-s001.zip › Fig. S4 Phylogenetic tree of the GP5 gene from 518 PRRSV strains inferred using the ML method in MEGA under the K2+G+I model, with 1000 bootstrap replicates.png]
